# Supplementary material for: Formation of metastable phases by spinodal decomposition
Source: Nat Commun. 2016 Oct 7;7:13067. doi: 10.1038/ncomms13067 (PMC5059762; doi:10.1038/ncomms13067)
Supplement: Supplementary Information — Supplementary Figure 1, Supplementary Note 1, Supplementary Discussion, Supplementary Methods and Supplementary References [file ncomms13067-s1.pdf]

## SUPPLEMENTARY FIGURE

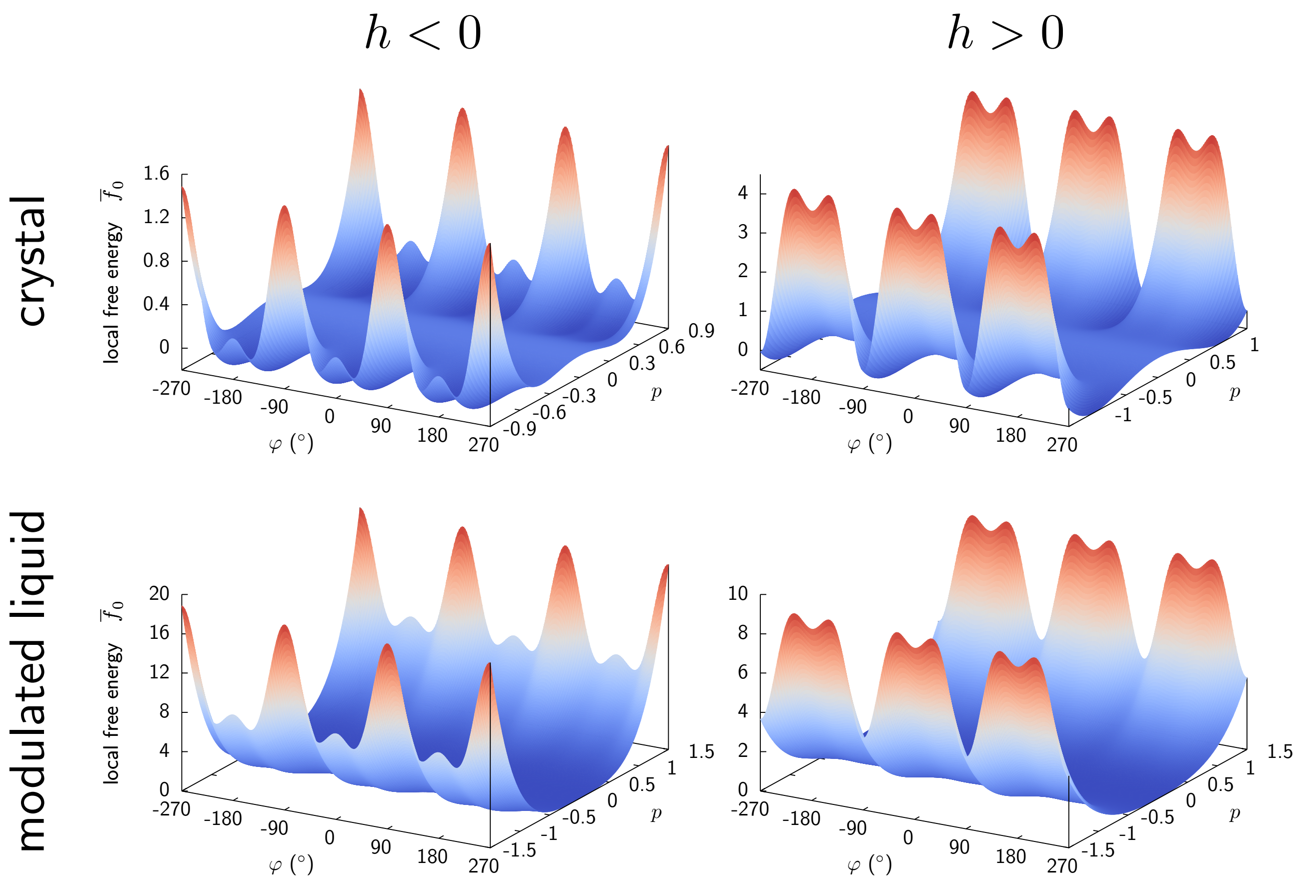


**Supplementary Figure** 1. Model free energy of the LIPT including liquid phases, Eq. 3, for $A = 2$ and $b = 6$. The four possible situations are shown, considering whether modulated liquid ($a = 1$) or solid ($a = -2$) phases are stabilized and whether a stable-metastable combination of crystals ($h = -2$) or two degenerated crystals ($h = 1$) are favoured. Liquid ($p = 0$) and crystalline ($p \neq 0$) phases are distinguished by the modulus $p$ of the vector order parameter $\mathbf{p}$ while the crystalline phases are described by the polar angle $\varphi$.

## SUPPLEMENTARY NOTE 1

### The inversion of the energy landscape as a probe for front propagation

In this note we speculate on the possibilities of employing the inversion of the energy landscape as an experimental test bench in which to study front propagation problems. The dynamics of a flat front (Eq. 5 in Methods),

$$-v\frac{d\varphi}{dz} = \Gamma\left(-\frac{\partial f_0}{\partial \varphi} + \kappa\frac{d^2\varphi}{dz^2}\right), \tag{1}$$

has a mechanical analogy to a particle moving under a one-dimensional potential $V(x)$, namely $m\ddot{x} = -\gamma\dot{x} - V'(x)$, with the order parameter $\varphi$ corresponding to the spatial coordinate $x$, and the normal coordinate $z$ corresponding to the time $t$. Both equations are completely analogous for a particle of mass $m \leftrightarrow \kappa$ moving under a potential $V \leftrightarrow -f_0$ and with a friction coefficient $\gamma \leftrightarrow v/\Gamma$. Hence, the motion of a flat interface under a given free energy $f_0$ can be mapped to the motion of a particle under the inverted potential $V \leftrightarrow -f_0$[1,2]. Therefore, since the LIPT realizes such an inverted potential, its dynamics could potentially be employed to experimentally probe front propagation problems via the described mechanical analogy. For instance, the dynamics at $H > H_s$ could in principle reveal information about the stable-metastable fronts propagating at $H < H_s$, which could be elusive to direct observation. The realization of these proposed tests remains an appealing challenge.

## SUPPLEMENTARY DISCUSSION

### Summary of the phase-ordering kinetics of the classical dynamical models and comparison to the LIPT scenario

This discussion gives a very brief summary of the phase-ordering kinetics associated to the classical dynamical models of phase transitions[3] (models A and B). Later, it also discusses the phase-ordering processes under inversion of the energy landscape for a system with a conserved order parameter. In addition to the results reported in the article, this allows a full comparison between the classical models and the LIPT scenario, as summarized in Fig. 5 in the article.

The initial-stage dynamics of phase separation depends on whether the system is at an unstable or a metastable state after the quench. In the first case, the system phase separates by the growth of long-wavelength fluctuations in a process generically known as spinodal decomposition. In the latter case, the system needs to overcome a free energy barrier to reach the stable state, which is done via a nucleation process. The domain formation process is thus determined by the coarse-grained free energy landscape. After forming, domains grow until phase separation is completed.

At late stages of phase separation, the evolution is dictated by the deterministic motion of sharp interfaces[4]. For a wide range of parameters, the dynamical field models mentioned above predict universal domain coarsening dynamics. For systems described by a scalar order parameter, the coarsening dynamics are completely specified by the dimensionality of the system and the presence or absence of a conservation law for the order parameter[5–7]. For nonconserved order parameters (model A[3]), coarsening follows the so-called Allen-Cahn law[8], while in systems with a conserved order parameter (model B[3]) domains grow following the Lifshitz-Slyozov law[9] in a process known as Ostwald ripening.

The results of this classical picture must be compared to the LIPT scenario. The article presents the results for our experimental case of a nonconserved order parameter, the lattice angle $\alpha$ of the colloidal crystals. The theoretical model of the LIPT allows to predict how these results would be modified in the case of a conserved order parameter.

First, upon a quench from $H > H_\mathsf{s}$ to $H < H_\mathsf{s}$, a conservation law for the order parameter would forbid the elimination of metastable states. Consequently, the fraction of spontaneously generated metastable phase remains constant. Clearly, this stabilization of the metastable state has a dynamical origin. Then, in this case, the stable and stabilized metastable domains would grow by the usual diffusive coarsening process (model B dynamics). Hence, the late-stage phase-ordering kinetics would feature the scaling regime known as the Lifshitz-Slyozov law[5–7,9,10] $R\left(t\right) \propto t^{1/3}$, where $R$ is the typical domain size.

In turn, following a quench from $H < H_\mathsf{s}$ to $H > H_\mathsf{s}$, a system with a conserved order parameter would always form two interfaces, since metastable domains are then dynamically stabilized at $H < H_\mathsf{s}$, and thus in permanent coexistence with the stable ones. Correspondingly, in this case, a diffusive instead of a curvature-driven coarsening would take place, again with the corresponding Lifshitz-Slyozov scaling[5–7,9,10] $R\left(t\right) \propto t^{1/3}$.

## SUPPLEMENTARY METHOD

### Model of the LIPT including liquid phases

The dynamical model introduced in the article, based on Eq. 1 in Methods, assumes that spatial inhomogeneities simply correspond to gradients of the scalar order parameter $\varphi$. However, this may not be the case in the experimental system, where the order parameter is the crystalline lattice angle $\alpha$. There, since rotational invariance is broken by the angular dependence of the energy, crystalline regions with nonequilibrium lattice angles are energetically penalized (Fig. 1 in the article). Hence, regions of different crystalline order $\alpha$ may not necessarily be connected via interfaces with a gradual change of the crystalline angle $\alpha$. Instead, liquid interfaces are likely to connect crystalline domains in the experiment.

We have incorporated this possibility into an extended model of the LIPT that is closer to the experimental situation. This model considers a 2D vector order parameter $\mathbf{p}\left(\mathbf{r},t\right)$ whose modulus $p\left(\mathbf{r},t\right)$ discriminates between crystalline and liquid phases while its polar angle $\varphi\left(\mathbf{r},t\right)$ indicates the lattice angle of the crystals as previously. Note that liquid phases are actually modulated liquids because of the underlying periodic potential of the substrate. Then, by means of a Landau expansion in the modulus $p\left(\mathbf{r},t\right)$, we write the simplest coarse-grained local free energy that allows for liquid phases:

$$f_\mathsf{L}\left[p\right] = \frac{a}{2}p^2 + \frac{b}{4}p^4. \qquad (2)$$

Here, $a < 0$ stabilizes the crystalline phase $p \neq 0$ while $a > 0$ stabilizes the modulated liquid phase $p = 0$. Note that the liquid phase does not have a well-defined value of the lattice angle $\varphi$. Also note that the proposed free energy does not correctly describe the liquid-solid transition, but it serves the purpose of allowing for liquid interfaces.

Now, in order to have liquid interfaces connecting two equilibrium crystalline states of the LIPT potential, we need to couple the liquid-solid transition to the LIPT. Nevertheless, this coupling must allow both transitions to be controlled

independently. The liquid-solid transition is controlled by the parameter $a$ in Eq. 2, while the LIPT is controlled by the landscape-inversion parameter $h$ (Eq. 2 in Methods). Therefore, these two parameters must remain decoupled, and the coupling must involve the always stabilizing $p^4$ term of Eq. 2 instead. Hence, the local free energy fulfilling these requirements reads

$$\overline{f}_0\left[\vec{p}\right] = f_{\mathsf{L}}\left[p\right] + \frac{p^4}{4} f_0\left[\varphi\right]. \tag{3}$$

This free energy is plotted in Supplementary Fig. 1 for the four possible situations regarding the liquid-solid transition and the landscape-inversion transition. Note that the nonzero value of $p$ for the solids depends on the crystalline order $\varphi$ and on $h$.

Finally, we must include the energetic contributions of the inhomogeneities of the vector field $\mathbf{p}\left(\mathbf{r},t\right)$. We model the free energy penalty of these distortions as that of a 2D nematic phase with elastic isotropy[11]:

$$f_{\mathsf{n}}\left[\mathbf{p}\right] = \frac{\kappa}{2}\partial_\alpha p_\beta \partial_\alpha p_\beta = \frac{\kappa}{2}\left[\left(\nabla p\right)^2 + p^2\left(\nabla\varphi\right)^2\right], \tag{4}$$

where $\kappa$ is the elastic constant. The same result is obtained from the hamiltonian of the continuum version of the classical XY model[7,12], then with $\kappa$ being the magnetic exchange interaction constant $J$. This completes the coarse-grained free energy functional for the LIPT extended to include the presence of liquid phases in the 2D experimental system:

$$F\left[\mathbf{p}\right] = u\int_\Omega \left(\overline{f}_0\left[\mathbf{p}\right] + f_{\mathsf{n}}\left[\mathbf{p}\right]\right) d^2\mathbf{r}, \tag{5}$$

where $u$ is an energy density scale as in Methods. Note that Eq. 5 reduces to Eq. 1 in Methods for a uniform nonzero modulus field $p\left(\mathbf{r},t\right)$.

Supplementary Fig. 1 shows that allowing for liquid regions preserves all the features of the phase-ordering processes of the LIPT discussed in the article, as obtained from the simplified model introduced in Methods. In particular, allowing for the more realistic liquid interfaces between crystals does not prevent the formation of two different interfaces between the two degenerated crystals appearing for $h < 0$. This can be clearly seen in the top left panel of Supplementary Fig. 1, where two different energy barriers can still connect the two degenerated equilibrium crystalline phases at $p \neq 0$. The system will in general form a combination of these two crystal interfaces and liquid interfaces through $p = 0$ according to their relative energetic cost.

In general, a model similar to the one above, allowing for the coexistence of liquid and several crystalline phases, could provide further insight into the kinetics of solid-solid phase transitions mediated by liquids[13].

## SUPPLEMENTARY REFERENCES


1. Desai, R. C. & Kapral, R. *Dynamics of self-organized and self-assembled structures* (Cambridge University Press, 2009).
2. Pismen, L. M. *Patterns and interfaces in dissipative dynamics* (Springer, 2006).
3. Hohenberg, P. & Halperin, B. Theory of dynamic critical phenomena. *Rev. Mod. Phys.* **49**, 435-479 (1977).
4. Cahn, J. W. & Hilliard, J. E. Free Energy of a Nonuniform System. I. Interfacial Free Energy. *J. Chem. Phys.* **28**, 258-267 (1958).
5. Langer, J. S. in *Solids far from equilibrium* (ed. Godrèche, C.) (Cambridge University Press, 1992).
6. Bray, A. J. Theory of phase-ordering kinetics. *Adv. Phys.* **43**, 357-459 (1994).
7. Chaikin, P. M. & Lubensky, T. C. *Principles of condensed matter physics* (Cambridge University Press, 1995).
8. Allen, S. M. & Cahn, J. W. A microscopic theory for antiphase boundary motion and its application to antiphase domain coarsening. *Acta Metall.* **27**, 1085-1095 (1979).
9. Lifshitz, I. M. & Slyozov, V. V. The kinetics of precipitation from supersaturated solid solutions. *J. Phys. Chem. Solids* **19**, 35-50 (1961).
10. Binder, K. Theory of first-order phase transitions. *Reports Prog. Phys.* **50**, 783-859 (1987).
11. de Gennes, P.-G. & Prost, J. *The physics of liquid crystals* (Oxford University Press, 1993).
12. Chowdhury, D. & Stauffer, D. *Principles of equilibrium statistical mechanics* (Wiley-VCH, 2000).
13. Peng, Y. et al. Two-step nucleation mechanism in solid-solid phase transitions. *Nat. Mater.* **14**, 101-108 (2015).
