# Supplementary figures and images for: Formation of metastable phases by spinodal decomposition
Source: Nat Commun. 2016 Oct 7;7:13067. doi: 10.1038/ncomms13067 (PMC5059762; doi:10.1038/ncomms13067)

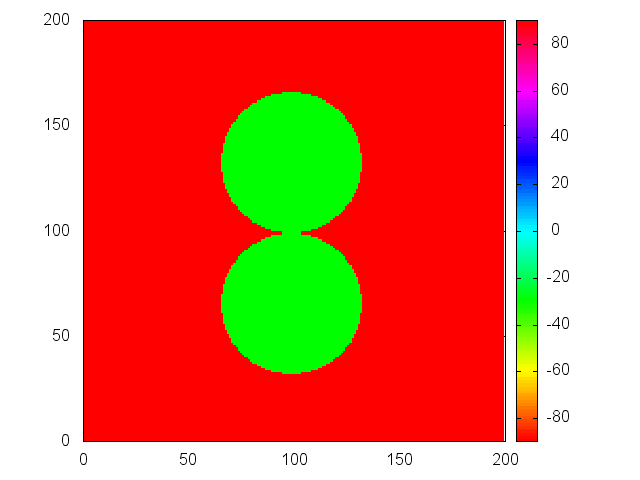

Supplement: Supplementary Movie 1 — Illustration of the formation of metastable domains by spinodal decomposition and their subsequent elimination by front propagation. [file ncomms13067-s2.gif]

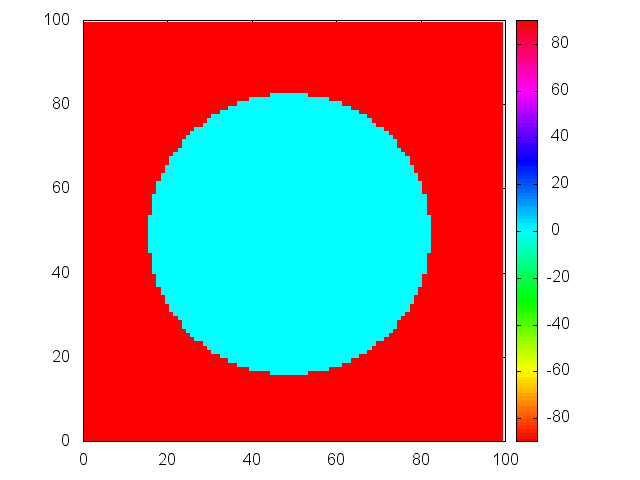

Supplement: Supplementary Movie 2 — Illustration of the coexistence of two equilibrium phases connected by two physically different interfaces. [file ncomms13067-s3.gif]

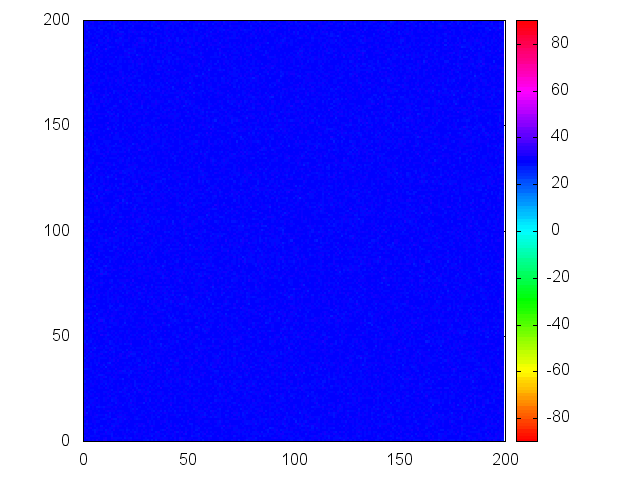

Supplement: Supplementary Movie 3 — Illustration of the formation of the least energetic interface when possible, even when the most energetic interface is initially present. [file ncomms13067-s4.gif]
